# Supplementary material for: The Re-Identification of Previously Unidentifiable Clinical Non-Tuberculous Mycobacterial Isolates Shows Great Species Diversity and the Presence of Other Acid-Fast Genera
Source: Pathogens. 2022 Oct 7;11(10):1159. doi: 10.3390/pathogens11101159 (PMC9610484; doi:10.3390/pathogens11101159)
Supplement: Supplementary file 1 [file pathogens-11-01159-s001.zip › pathogens-1891036-supplementary.pdf]

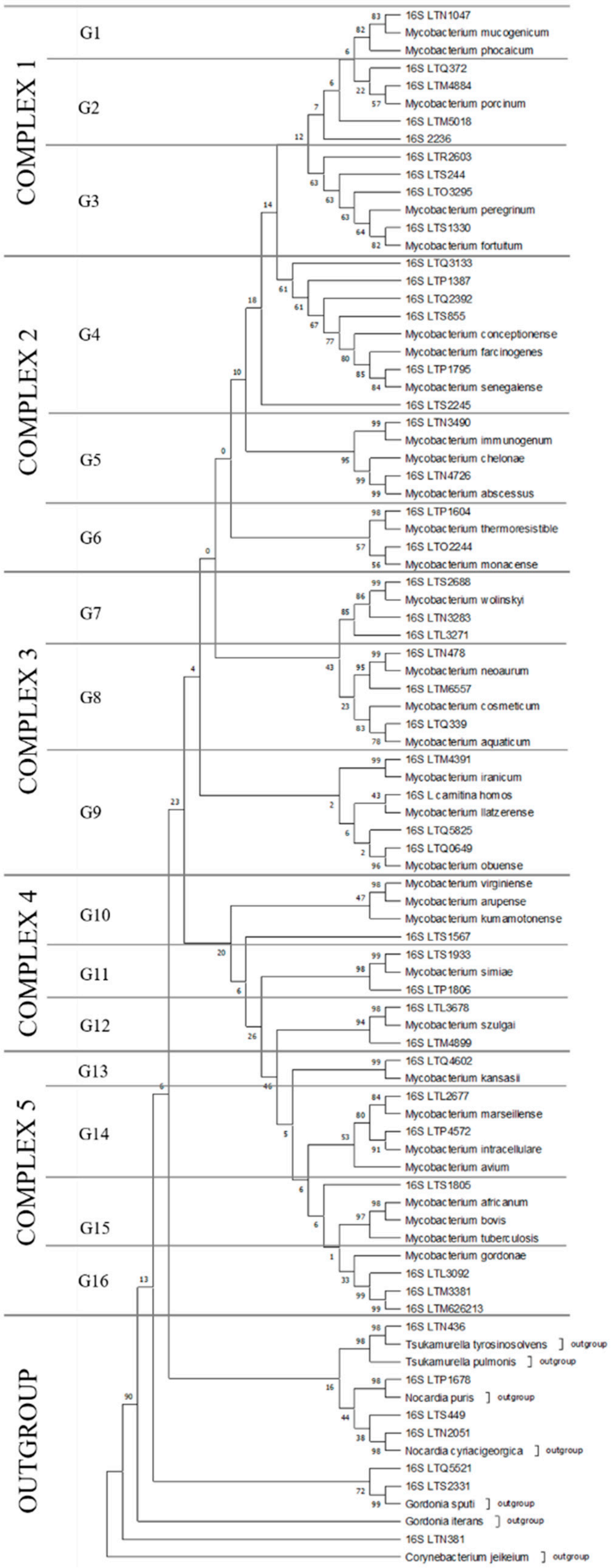


**Figure S1.** Phylogenetic tree of *16S rRNA* sequences of the mycobacterium reference sequences, outgroup strains and the *Mycobacterium* spp. strains from this study.

**Table S1.** Origin and identification of acid-fast bacteria, identified as *Mycobacteria* with the ZN staining. Shown are the identification with PRA, and identification with BLAST with the sequence of the *16S* gene. The strains LTR2603, LTS244, LTO3295, and LTN4726 were identified with the *hsp65* sequence, and the strains LTP1387, LTQ2392, and LTS855 with the *rpoB* sequences. The final column is the species identification based on a phylogenetic analysis.

| | Groups | Code[a] | Year[b] | Age | Sex | Sample Type[c] | Pre-identification with PRA[d] | Blast Identification: 16S, rpoB and/or Hsp65 Sequence | Species identification by phylogenetic Analysis |
|---|---|---|---|---|---|---|---|---|---|
| **COMPLEX 1** | G1 | LTN1047 | 2016 | 39 | F | Secretion | *M. spp.* | *M. mucogenicum* | *M. mucogenicum* and *phocaicum* [e] |
| | G2 | LTQ372 | 2019 | 28 | F | NA[f] | *M. abscessus* | *M. phocaicum* | *M. porcinum* [e] |
| | | LTM4884 | 2015 | NA | F | NA | *M. spp.* | *M. porcinum/M. fotuitum* | *M. porcinum* |
| | | LTM5018 | 2015 | 86 | M | Sputum | *M. spp.* | *M. phocaicum/M. mucogenicum* | *M. porcinum* |
| | | LTN2236 | NA | NA | NA | Tap water medical consulting room | *M. spp.* | *M. phocaicum/M. mucogenicum* | *M. porcinum* |
| | G3 | LTR2603 | 2021 | 74 | M | Secretion | *M. tuberculosis/ M. fortuitum* | *M. fortuitum/M. spp./M. gilvum* | *M. fortuitum* |
| | | LTS244 | 2018 | 70 | M | Secretion | *M. tuberculosis/ M. fortuitum* | *M. fortuitum M. fortuitum/M. spp./M. gilvum* | *M. fortuitum* |
| | | LTO3295 | 2017 | 45 | F | Sputum | *M. spp.* | *M. fortuitum M. fortuitum/M. spp./M. gilvum* | *M. fortuitum* |
| | | LTS1330 | 2021 | 63 | F | Sputum | NA | *M. fortuitum M. fortuitum/M. spp./M. gilvum* | *M. fortuitum* |
| **COMPLEX 2** | G4 | LTQ3133 | 2019 | 42 | M | Biopsy | *M. peregrinum* | *M. senegalense/M. conceptionense/M. farcinogenes/M. houstonense/M. fortuitum* | *M. conceptionense, senegalense* and *farcinogenes* [e] |
| | | LTP1387 | 2018 | 62 | F | Pleural fluid | *M. spp.* | *M. senegalense/M. conceptionense/M. farcinogenes/M. houstonense/M. fortuitum* | *M. senegalense* and *farcinogenes* [e] |
| | | LTQ2392 | 2019 | 24 | F | Secretion | *M. peregrinum* | *M. senegalense/M. conceptionense/M. farcinogenes/M.* | *M. conceptionense* |

| | | | | | | | | | |
|---|---|---|---|---|---|---|---|---|---|
| | | | | | | | | *houstonense/M. fortuitum* | |
| | | LTS855 | 2021 | 19 | F | Sputum | *M. tuberculosis/ M. fortuitum* | *M. senegalense/M. conceptionense/M. farcinogenes/M. houstonense/M. fortuitum* | *M. conceptionense* |
| | | LTP1795 | 2018 | 41 | F | Secretion | *M. spp.* | *M. senegalense/M. conceptionense/M. farcinogenes/M. houstonense/M. fortuitum* | *M. conceptionense, senegalense* and *farcinogenes* [e] |
| | | LTS2245 | 2021 | 68 | M | Sputum | *M. spp.* | *M. senegalense/M. conceptionense/M. farcinogenes/M. houstonense/M. fortuitum* | *M. conceptionense, senegalense* and *farcinogenes* [e] |
| | G5 | **LTN3490** | 2016 | 60 | M | NA | *M. spp.* | M. immunogenum/ M. chelonae/M. spp. | *M. inmmunogenum abscesus* and *chelonae* [e] |
| | | LTN4726 | 2016 | 17 | M | Biopsy | *M. spp.* | M. chelonae/M. *abscesus* | *M. abscessus* |
| | G6 | LTP1604 | 2018 | 58 | M | Pleural fluid | *M. spp.* | *M. thermoresistibile/ M. spp.* | *M. thermoresistibile* |
| | | LTO2244 | 2017 | 45 | F | Sputum | *M. spp.* | N. cyriacigeorgica/ M. vanbaalenii | Non-identifiable |
| **COMPLEX 3** | G7 | LTS2688 | 2021 | 8 | M | Pleural fluid | NA | M. wolinskyi/M. jacuzzii | *M. wolinskyi* |
| | | LTN3283 | 2016 | - | - | Sauce | *M. goodii/M. smegmatis* | M. wolinskyi/M. jacuzzii | *M. wolinskyi* |
| | | LTL3271 | 2014 | 27 | F | Secretion | *M. spp.* | M. jacuzzii/M. peregrinum/M. spp. | *M. wolinskyi* |
| | G8 | LTN478 | 2016 | 43 | F | Sputum | *M. spp.* | *M. bacteremicum/M. neoaurum/M. spp.* | *M. neoaurum* |
| | | LTM6557 | 2015 | 77 | F | Sputum | *N. spp./M. neonarum* | M. bacteremicum/ M. neoranum | *M. neoaurum* |
| | | LTQ339 | 2019 | 19 | M | Pleural fluid | *M. spp.* | *M. cosmeticum/M. spp./M. wolinskyi* | *M. cosmeticum* |

| | | | | | | | | | |
|---|---|---|---|---|---|---|---|---|---|
| | G9 | **LTM4391** | 2015 | 40 | M | Secretion | *M. spp.* | *M. iranicum/M. spp.* | *M. iranicum* |
| | | L-carnitine | NA | - | - | Cosmetic product | *M. mucogenicum* | *M. mucogenicum/M. phocaicum/M. spp.* | *M. llatzerense* |
| | | **LTQ5825** | 2019 | 48 | F | Secretion | *N. spp.* | *M. mucogenicum/M. phocaicum/M. spp./M. Llatzerense* | *M. llatzerense* [e] |
| | | LTQ0649 | 2019 | 30 | F | Pleural fluid | *Actinomicetae* | *M. obuense/M. spp./M. kyogaense* | *M. obuense* |
| **COMPLEX 4** | G10 | LTS1567 | 2021 | 63 | F | Sputum | NA | *M. crocinum/M. spp./M. gilvum/M. chitae/M. aquaticum* | Non-identifiable |
| | G11 | LTS1933 | 2021 | 63 | F | Sputum | NA | *M. simiae* | *M. simiae* |
| | | LTP1806 | 2018 | 65 | F | Pleural fluid | *M. spp.* | *M. simiae* | *M. simiae* |
| | G12 | LTL3678 | 2014 | 26 | M | Sputum | *N. spp.* | *M. szulgai/M. angelicum* | *M. szulgai* |
| | | LTM4899 | 2015 | 79 | M | Pleural fluid | *N. spp.* | *M. szulgai/M. angelicum/M. spp.* | *M. szulgai* |
| **COMPLEX 5** | G13 | LTQ4602 | 2019 | 66 | M | Sputum | NA | *M. kansasii/M. gastri/M. ostraviense* | *M. kansasii* |
| | G14 | LTL2677 | 2014 | 59 | F | Sputum | *M. spp.* | *M. marseillense/M. yongonense/M. intracellulare* | *M. marseillense, intracellulare y avium* [e] |
| | | LTP4572 | 2018 | 76 | M | Sputum | *M. chimaera* | *M. paraintracellulare /M. intracellulare subspp. chimaera* | *M. marseillense, intracellulare y avium* [e] |
| | G15 | LTS1805 | 2021 | 58 | F | Secretion | NA | *M. ulcerans/M. marinum* | Non-identifiable |
| | G16 | **LTL3092** | 2014 | 24 | F | Biopsy | *N. spp.* | *C. jeikeium/C. amycolatum* | Non-identifiable |
| | | LTM3381 | 2015 | 61 | F | Urine | *N. spp.* | *C. jeikeium* | Non-identifiable |
| | | **LTM6213** | 2015 | 82 | F | Sputum | *M. chimaera/M. intracellulare* | *C. jeikeium/C. amycolatum* | Non-identifiable |
| | Outgroup | LTN436 | 2016 | 43 | F | Sputum | *M. spp.* | *T. pulmonics/T. conjunctivitidis* | *T. pulmonis* |

| | | | | | | | |
|---|---|---|---|---|---|---|---|
| LTP1678 | 2018 | 70 | F | Sputum | *Actinomiceta e* | *N. puris/N. spp.* | *N. puris* |
| LTS449 | 2021 | 62 | F | Secretion | *N. spp.* | *N. niwae/N. beijingensis* | *N. cyriacigeorgica* [e] |
| LTN2051 | 2016 | - | M | Sputum | *M. spp.* | *N. cyriacigeorgica/N . spp.* | *N. cyriacigeorgica* |
| LTQ5521 | 2019 | 29 | M | Pleural fluid | *M. abscessus* | *G. bronchialis/G. spp./Actinomycet ia bacterium* | Non-identifiable |
| LTS2331 | 2021 | 30 | M | Sputum | *N. spp.* | *G. sputi/G. spp.* | *G. sputi* |
| LTN381 | 2016 | 64 | M | Pleural fluid | *M. spp.* | *Clostridium perfringens* | Non-identifiable |

[a] Laboratory code Caracas; [b]Year of isolation; [c]Type of clinical sample; [d]Pre-identification by PRA;.
[e]Near (NR);.
NA = not available. These are patients' samples but unknow is the type of clinical sample.
Genera: M.: *Mycobacterium*, N*: *Nocardia*, T*: *Tsukamurella*, G*: *Gordonia*; C*: *Corynebacterium*. For strains LTM4391, LTM6213, LTN3490, LTL3092 and LTQ5825, indicated in bold red in the table, no PCR product for the hsp65 PCR was obtained.

**Table S2.** GenBank accession numbers of *Mycobacterium* species sequences used in the phylogenetic analysis.

| | ID Genbank | | |
|---|---|---|---|
| ***Mycobacterium* Species** | ***16S*** | ***rpoB*** | ***hsp65*** |
| *M. bovis* | AF547903 | AF057451 | AF547813 |
| *M. africanum* | AF547893 | JF923622 | AF547803 |
| *M. tuberculosis* | X52917 | L27989 | AY299144 |
| *M. avium* Sub. Avium | AJ536037 | - | - |
| *M. abscessus* | AY457071 | AY262741 | AY458075 |
| *M. cosmeticum* | AY449728 | DQ124109 | AY449730 |
| *M. fortuitum* | AY457066 | AY147165 | AY458072 |
| *M. wolinskyi* | AY457083 | - | - |
| *M. obuense* | X55597 | AY544951 | - |
| *M. arupense* | DQ157760 | JN571215 | EU191917 |
| *M. monacense* | AF107039 | EU191923 | JF491320 |
| *M. neoaurum* | AF480593 | JF712875 | AF547860 |
| *M. gordonae* | X52923 | AY544919 | AF547840 |
| *M. mucogenicum* | AY457074 | AY147171 | AY458078 |
| *M. szulgai* | X52926 | AY544966 | AF547878 |
| *M. phocaicum* | AY859682 | AY859693 | DQ987726 |
| *M. kansasii* | AJ536035 | KY933079 | AF547849 |
| *M. conceptionense* | AY859684 | AY859695 | AM902957 |
| *M. senegalense* | AY457081 | AY262738 | AY458067 |
| *M. virginiense* | KR025879 | KR025893 | MK587449 |
| *M. kumamotonense* | JN571176 | JN571253 | AB239920 |
| *M. thermoresistibile* | X55602 | AY544968 | - |
| *M. marseillense* | EU266632 | EF584444 | EU239788 |
| *M. intracellulare* | AJ536036 | JQ411539 | AF547848 |
| *M. iranicum* | HQ009482 | - | - |
| *M. farcinogenes* | AY457084 | AY262742 | AF547830 |
| *M. porcinum* | AF480588 | AY544955 | AF547867 |
| *M. peregrinum* | AF547956 | AY147166 | AY458069 |

| | | | |
|---|---|---|---|
| *M. chelonae* | AF547909 | AY147163 | JX154110 |
| *M. simiae* | AF547966 | AY544963 | AF547875 |
| *M. aquaticum* | KY392537 | - | - |
| *M. llatzerense* | AJ746070 | - | - |
| *M. immunogenum* | HE654001 | - | - |
| *Nocardia puris* | AJ508748 | DQ08514 | AY903632 |
| *Nocardia cyriacigeorgica* | KP137526 | DQ085116 | AY756522 |
| *Tsukamurella pulmonis* | X92981 | KX932012 | - |
| *Tsukamurella tyrosinosolvens* | Y12248 | KX932015 | - |
| *Corynebacterium jeikeium* | AB470615 | AY492231 | - |
| *Gordonia iterans* | AB624257 | - | - |
| *Gordonia sputi* | X81923 | - | - |

**Table S3.** DNA Models test, divergence percentages, and number of identifications for each gene.

| | | | % DIVERGENCE | | | |
|---|---|---|---|---|---|---|
| **Gene** | **Model** | **Outgroup** | **Ingroup** | **Between** | **Overall** | **Identifications** |
| ***16S*** | T92[a]+G+I | 2.99E+05 | 1.328 | 1-9 | 4.631 | 22 |
| ***rpoB*** | GTR[b] +G+I | - | 5.068 | 5.75-13.61 | 9.232 | 2 |
| ***hsp65*** | GTR+G+I | - | 3.606 | 2.32-12.48 | 8.288 | 5 |

[a]:Tamura 3-parameter is the estimation of the number of nucleotide substitutions when there are strong transition-transversion and G + C-content biases; [b]: General Time Reversible gives nucleotides consisting of parameters of an equilibrium base frequency vector, where frequency at each base occurred at this site and rate matrix.

**Table S4.** *16S* rRNA sequences of the 48 strains that were identified with ZN staining as belonging to the genus *Mycobacteria*.

| **#Accession** | **Sequence ID** |
|---|---|
| ON171319 | Mycobacterium1 |
| ON171320 | Mycobacterium2 |
| ON171321 | Mycobacterium3 |
| ON171322 | Nocardia4 |
| ON171323 | Corynebacterium5 |
| ON171324 | Mycobacterium6 |
| ON171325 | Gordonia7 |
| ON171326 | Mycobacterium8 |
| ON171327 | Mycobacterium9 |
| ON171328 | Mycobacterium10 |
| ON171329 | Mycobacterium11 |
| ON171330 | Tsukamurella12 |
| ON171331 | Mycobacterium13 |
| ON171332 | Nocardia14 |
| ON171333 | Mycobacterium15 |
| ON171334 | Mycobacterium16 |
| ON171335 | Mycobacterium17 |
| ON171336 | Gordonia18 |
| ON171337 | Mycobacterium19 |
| ON171338 | Mycobacterium20 |
| ON171339 | Mycobacterium21 |
| ON171340 | Mycobacterium22 |
| ON171341 | Mycobacterium23 |

| | |
|---|---|
| ON171342 | Mycobacterium24 |
| ON171343 | Mycobacterium25 |
| ON171344 | Corynebacterium26 |
| ON171345 | Mycobacterium27 |
| ON171346 | Mycobacterium28 |
| ON171347 | Clostridium29 |
| ON171348 | Mycobacterium30 |
| ON171349 | Mycobacterium31 |
| ON171350 | Mycobacterium32 |
| ON171351 | Mycobacterium33 |
| ON171352 | Mycobacterium34 |
| ON171353 | Mycobacterium35 |
| ON171354 | Mycobacterium36 |
| ON171355 | Nocardia37 |
| ON171356 | Mycobacterium38 |
| ON171357 | Mycobacterium39 |
| ON171358 | Corynebacterium40 |
| ON171359 | Mycobacterium41 |
| ON171360 | Mycobacterium42 |
| ON171361 | Mycobacterium43 |
| ON171362 | Mycobacterium44 |
| ON171363 | Mycobacterium45 |
| ON171364 | Mycobacterium46 |
| ON171365 | Mycobacterium47 |
| ON171366 | Mycobacterium48 |
